# Are white matter hyperintensities associated with neuroborreliosis? The answer is twofold

**DOI:** 10.1007/s00234-024-03482-0

**Published:** 2024-10-18

**Authors:** Elisabeth S. Lindland, Martin S. Røvang, Anne Marit Solheim, Silje Andreassen, Ingerid Skarstein, Nazeer Dareez, Bradley J. MacIntosh, Randi Eikeland, Unn Ljøstad, Åse Mygland, Steffan D. Bos, Elling Ulvestad, Harald Reiso, Åslaug R. Lorentzen, Hanne F. Harbo, Atle Bjørnerud, Mona K. Beyer

**Affiliations:** 1 Department of Radiology, Sorlandet Hospital, Sykehusveien 1, 4838 Arendal, Norway; 2 Institute of Clinical Medicine, University of Oslo, Oslo, Norway; 3 Department of Physics and Computational Radiology, Oslo University Hospital, Oslo, Norway; 4 Department of Neurology, Sorlandet Hospital, Kristiansand, Norway; 5 Institute of Clinical Medicine, University of Bergen, Bergen, Norway; 6 Department of Pediatrics, Sorlandet Hospital, Arendal, Norway; 7 Department of Microbiology, Haukeland University Hospital, Bergen, Norway; 8 The Norwegian National Advisory Unit on Tick-Borne Diseases, Sorlandet Hospital, Kristiansand, Norway; 9 Faculty of Health and Sport Sciences, University of Agder, Kristiansand, Norway; 10 Cancer Registry of Norway, The Norwegian Institute of Public Health, Oslo, Norway; 11 Department of Neurology, Oslo University Hospital, Oslo, Norway; 12 Department of Physics, University of Oslo, Oslo, Norway; 13 Division of Radiology and Nuclear Medicine, Oslo University Hospital, Oslo, Norway

**Keywords:** Magnetic resonance imaging, Central nervous system infections, Lyme neuroborreliosis, Tick-borne diseases

## Abstract

**Purpose:**

Many consider white matter hyperintensities (WMHs) to be important imaging findings in neuroborreliosis. However, evidence regarding association with WMHs is of low quality. The objective was to investigate WMHs in neuroborreliosis visually and quantitatively.

**Materials and methods:**

Patients underwent brain MRI within one month of diagnosis and six months after treatment. Healthy controls were recruited. WMHs were counted by visual rating and the volume was calculated from automatic segmentation. Biochemical markers and scores for clinical symptoms and findings were used to explore association with longitudinal volume change of WMHs.

**Results:**

The study included 74 patients (37 males) with early neuroborreliosis and 65 controls (30 males). Mean age (standard deviation) was 57.4 (13.5) and 57.7 (12.9) years, respectively. Baseline WMH lesion count was zero in 14 patients/16 controls, < 10 in 36/31, 10–20 in 9/7 and > 20 in 13/11, with no difference between groups (*p* = 0.90). However, from baseline to follow-up the patients had a small reduction in WMH volume and the controls a small increase, median difference 0.136 (95% confidence interval 0.051–0.251) ml. In patients, volume change was not associated with biochemical or clinical markers, but with degree of WMHs (*p* values 0.002–0.01).

**Conclusion:**

WMH lesions were not more numerous in patients with neuroborreliosis compared to healthy controls. However, there was a small reduction of WMH volume from baseline to follow-up among patients, which was associated with higher baseline WMH severity, but not with disease burden or outcome. Overall, non-specific WMHs should not be considered suggestive of neuroborreliosis.

**Supplementary Information:**

The online version contains supplementary material available at 10.1007/s00234-024-03482-0.

## Introduction

Lyme neuroborreliosis is a spirochetal tick-borne infection, which typically presents with painful radiculitis, meningitis and/or cranial neuropathy. However, the presentation can vary with a wide range of possible manifestations, such as encephalitis, vasculitis, myelitis, and peripheral neuropathy [1]. Diagnostic criteria stress the exclusion of other explanations for the clinical symptoms [2, 3]. Therefore, imaging examinations often contribute in the diagnosis and follow-up of patients with neuroborreliosis.

Initial imaging studies contributed to establish a theory that white matter hyperintensity (WMH) lesions are an important imaging feature of neuroborreliosis [4–10]. These studies conducted between 1988 and 2007 have several shortcomings. Firstly, they contained small sample sizes (*N* = 10–27). Secondly, generalizability of the results may be limited because diagnostic criteria have evolved later, and so the studies’ inclusion criteria were variable. Additionally, knowledge of the frequency and impact of white matter changes in adults has increased over time, which may alter interpretation of study results. More recent publications have larger sample sizes (*N* = 16–131) and do not report findings of demyelination or a higher frequency of white matter lesions in patients with neuroborreliosis compared to controls [11–14]. However, the largest study used non-standardized clinical radiology reports and did not perform a systematic evaluation of the images. None of the published studies have applied quantitative measurement methods for evaluation of white matter lesions. A previous study of white matter structure integrity with diffusion tensor imaging did not report alterations in patients (*N* = 20) compared to controls (*N* = 11) [4], and a recent case–control study (*N* = 12 + 18) also used diffusion tensor imaging for evaluation of white matter, but the patients had post-treatment Lyme disease syndrome, and are therefore not representative for patients with neuroborreliosis [15].

Recently, clinical practice guidelines have been published by the American societies for neurology, rheumatology and infectious diseases, and they suggest not to test for Lyme disease in the case of non-specific WMHs as an isolated finding. However, they acknowledge that their recommendation is weak and that evidence is of low quality [2]. There is a paucity of higher quality studies with prospective inclusion, longitudinal or case–control design and systematic, quantitative and reliable image analysis. We conducted a prospective longitudinal case–control study with the primary aim to test two hypotheses: 1) WMH lesion count is higher in patients with neuroborreliosis compared to healthy controls; 2) The total volume of WMHs and/or the change in volume of WMHs over time is different in patients compared to controls. The secondary aim was to describe the frequency of MR imaging abnormalities of the brain in a neuroborreliosis cohort with a standardized and systematic image evaluation method.

## Methods

### Participants

**Fig. 1:**
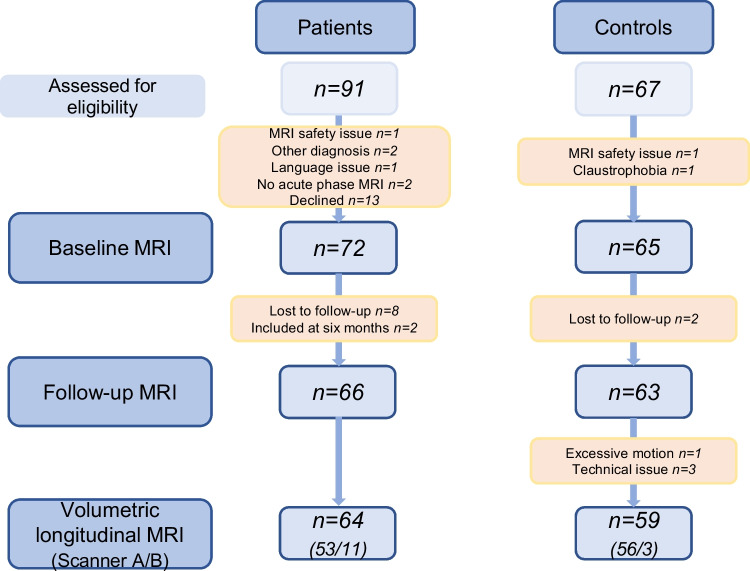
Flow chart of sample selection. The patients came to baseline MRI within one month of diagnosis and treatment initiation, and follow-up MRI was done six months after completion of antibiotic treatment. Two MRI scanners were used (**A** and **B**), and the number of participants per scanner is provided for the data used in volumetric analysis

Consecutive patients were invited to participate upon diagnosis with neuroborreliosis according to the European Federation of Neurological Societies’ diagnostic guidelines [3]. The criteria are 1) neurological symptoms suggestive of neuroborreliosis that are not explained by other causes, 2) cerebrospinal fluid (CSF) pleocytosis, and 3) intrathecal production of *Borrelia Burgdorferi (Bb)* specific antibody. The diagnosis is definite when all three criteria are fulfilled, and possible if two criteria are fulfilled. In clinical practice, the latter group mainly consists of individuals with pleocytosis and negative *Bb* antibody index. These cases are considered probable neuroborreliosis due to the *Bb* antibody index test’s lack of sensitivity in the early disease phase [16]. In this study, only patients with pleocytosis were included so all cases were definite or probable neuroborreliosis. Healthy controls were recruited by encouraging the patients to invite social contacts, and via newspaper advertisement. Controls were selected to match the patient´s sex and age ± two years. The inclusion period was November 2015–December 2018 for the patients, and August 2016–March 2019 for the controls. The study is part of a research project with subject overlap between a randomized clinical trial, MRI, and neuropsychology studies [12, 17–22]. A flow chart of sample selection is shown in Fig. 1.

### Imaging, visual rating and volumetry

Whole brain MRI examination was done within one month after diagnosis and treatment initiation, and the follow-up scan was done six months after treatment. Controls were scanned twice with the same interval. Scanners at different locations (320 km apart) were used to enhance participation with possibility of a scanner in reasonable travelling distance for the participants. There is a trade-off in study power as it is increased with higher sample size, but is reduced due to more technical variability. Two different 3 T MRI scanners were used, a Skyra system (Siemens, scanner A) with a 64-channel head coil and a Signa system (General Electric, scanner B) with a 32-channel head coil. Individuals had both scans on the same system, and sex- and age-matched control subjects were recruited with respect to the patients on each scanner system. For this current study on WMH assessment, we used the 3D fluid attenuated inversion recovery (FLAIR) images. From the baseline time point and for patients only, we also used contrast-enhanced fat saturated T1 weighted images ten minutes after gadoterate meglumine (Dotarem®, Guerbet) 0.5 mmol/ml 0.2 ml/kg as part of the assessment of imaging abnormalities. The scan parameters on the Skyra/Signa for FLAIR were: Slice thickness 0.48/1.2 mm, FOV 245/256 mm, TR 5000/8000 ms, TE, 389/90 ms, TI 1800/2071 ms, averages 1.0/1.0, and parameters for fat saturated T1 were: Slice thickness 0.93/1.0 mm, FOV 256/256 mm, TR 500/650 ms, TE 3.8/minimum ms, averages 1.0/1.0.

WMH lesion count was categorized by the following intervals: No lesions, < 10 lesions, 10–20 lesions, or > 20 lesions. Additionally, the commonly used Fazekas scale for grading of WMHs was applied [23]. Presence of encephalitic pattern, mass lesion, gadolinium enhancing brain lesion and/or leptomeningeal enhancement was rated with binary score, yes or no. Incidental findings or findings from other clinical imaging examinations were registered. To provide data for reliability, two neuroradiologists provided scores for a subset of the data (*N* = 69). Discrepant scores were re-read in consensus. One rater scored the remainder.

**Fig. 2:**
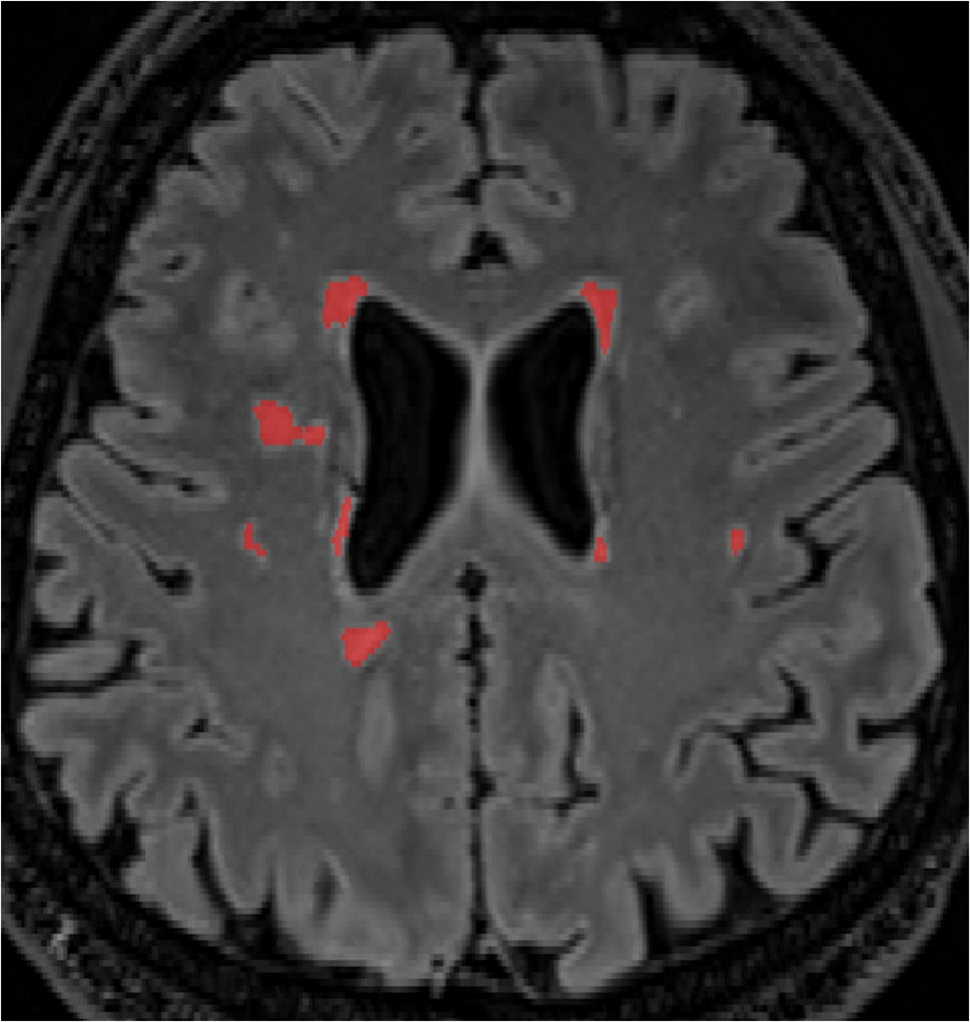
Automatic segmentation example case. Red voxels are automatically generated WMH lesion segmentation overlayed on axial FLAIR image

Volumetric analysis of WMHs was done with a deep learning segmentation model (3D-nnU-Net) [24]. Preprocessing included intensity bias-correction, intensity values below zero were set to zero, intensity z-normalization and resampling to isotropic voxels of 1 mm^3^. Probability output was thresholded at 0.5 to produce binary lesion classification mask. In short, the model was trained on 441 participants that were part of a national, multicenter study including healthy controls and individuals with mild cognitive impairment. The training data were external from the current groups and scanners. The model reported state-of-art WMH segmentation performance in volumetric FLAIR images. The segmentation outputs from 24 participants were edited in ITK-SNAP version 3.6.0 [25] by two neuroradiologists for investigation of reliability. For the whole study sample, segmentation output was visually checked for errors by one rater. Example case showing the automatically generated segmentation overlayed on the FLAIR image is given in Fig. 2. Imaging data were registered using the FMRIB Software Library (FSL; flirt tool) to a standard space atlas to facilitate visualization of the segmentation results in a common coordinate system. The binary segmentation masks were overlaid to produce an average lesion location map for each group and session.

### Clinical and laboratory data

Selected clinical and laboratory data from the patients were used as secondary endpoints in explorative analysis of correlation with the longitudinal volume change of WMHs. These data were collected at inclusion before treatment, and after six months. Scoring of subjective symptoms and objective clinical findings were summarized in a composite clinical score (CCS) as described for the clinical trial [21, 22]. This, as well as CSF cell count, total protein, and serum neurofilament light chain (sNfL), as measured by the bead-based immunoassay Simoa NF-light V2 Advantage Kit from Quanterix Corporation (Billerica, USA), were not available from the controls. Fatigue severity scale [26] was collected at both sessions for both groups.

### Statistics

Agreement of visual rating was analyzed with percentage of agreement and weighted kappa. Reliability of the WMH volumetry was analyzed with intra-class correlation coefficient (ICC) using a two-way mixed effects model, mean of raters type and absolute agreement definition [27], as well as Dice similarity coefficient (DSC) [28].

χ^2^, Student *t* and Mann–Whitney *U* tests were used for group comparisons, and Spearman *ρ* for bivariate correlation. Tests were two-tailed and for independent samples. Non-parametric tests were used for analyses that involved the volumetric data, as these were skewed towards low values. Due to the exploratory nature of the correlation analyses, no correction was performed for multiple comparisons. A multiple linear regression model was used to assess how well the variables baseline WMH volume, sNfL, and baseline CCS predict the WMH volume change in patients, after controlling for the influence of age, which is especially known to be associated with degree of WMHs as well as levels of sNfL [29, 30].

The statistical significance level of *p* = 0.05 was chosen. Participants with missing data for secondary endpoints were not excluded from analyses. Analyses were performed using SPSS version 29.

## Results

### Participants

In total, there were 74 patients and 65 control subjects with MRI scan from one or both assessment time points. Clinical trial participants (*n* = 63) were randomized, and received two or six weeks of oral doxycycline, and the remaining participants received oral doxycycline (1/11), initial intravenous ceftriaxone/penicillin followed by oral doxycycline (5/11) or intravenous ceftriaxone (5/11). There was no difference in sex distribution between the groups, odds ratio for patient male was 1.17 (95% confidence interval(CI) 0.60–2.27), (continuity corrected χ^2^ = 0.080, *p* = 0.78, *φ* = 0.038). Mean age (SD, range) was similar in the groups, 57.4 (13.5, 20.5–81.8) years for the patients, and 57.7 (12.9, 25.7–81.3) years for the controls, mean difference was 0.23 (95% CI -4.68–4.21) years (*t* = -0.10, *p* = 0.92, Cohen’s d = -0.017). Further patient characteristics are provided in Table 1.

**Table 1:** Characteristics of the participants

	Patients (*n* = 74)	Controls (*n* = 65)
Sex (N male/female)	37/37	30/35
Age, years	57.4 (13.5, 20.5–81.8)	57.7 (12.9, 26–81)
Duration neurological symptoms, days	28 (32, 1–180)	
Interval lumbar puncture – baseline MRI, days	16 (8, 4–31)	
CSF cells/mm^3^	188 (172, 7–752)	
CSF protein (g/l)	1.37 (0.83, 0.32–3.86)	
*Bb* antibody index positive (N)^a^	56	
sNfL (pg/ml)	53.0 (61.1, 2.6–315.4)	

*Bb* *Borrelia Burgdorferi*, *CSF* Cerebrospinal fluid, *sNfL* serum neurofilament light chain

^a^Thirteen patients had negative *Bb* antibody index. Their mean duration of symptoms was 14 days (SD 14, range 4–55), and therefore are cases of probable neuroborreliosis. *Bb* antibody index was missing for five patients

Values are mean (standard deviation, range)

Sixty-four patients and 59 controls had complete data from both time points for volumetric analysis of WMHs, cf. flow chart in Fig. 1. Among these patients, 53 were scanned on the Skyra and 11 on the Signa. For controls, the respective numbers were 56 on the Skyra and three on the Signa. The mean interval (SD, range) between scans for these patients was 211 days (20, 154–297), and for controls 208 days (16, 164–259). There was no statistically significant difference between the groups with respect to this interval as mean difference (95% CI) was 2.27 (-4.08–8.63) days (*t* = 0.71, *p* = 0.45, Cohen’s d = 0.13).

### WMH count, Fazekas scale and volume

WMH lesion count or Fazekas scale grade did not differ between the groups at baseline or follow-up. The numbers of patients and controls in each category of WMH lesion count are given in Table 2. Odds ratios at baseline/follow-up for patients vs. controls with no lesions as reference were 0.76 (95% CI 0.32–1.79)/0.69 (95% CI 0.28–1.68) for < 10 lesions, 0.68 (95% CI 0.20–2.31)/0.63 (95% CI 0.19–2.16) for 10–20 lesions and 0.74 (95% CI 0.25–2.17)/0.89 (95% CI 0.29–2.78) for > 20 lesions (χ^2^ = 0.57, *p* = 0.90, Cramer’s V = 0.064 and χ^2^ = 0.94, *p* = 0.82, Cramer’s V = 0.085, respectively). The proportions and group comparison results of Fazekas scale grade are provided in supplemental table S3.

**Table 2:** Contingency tables for white matter hyperintensity lesion count at baseline and follow-up

Lesion count		0	< 10	10*–*20	> 20	Total
Baseline	Patients	14 (46.7%)	36 (53.7%)	9 (56.3%)	13 (54.2%)	72 (52.6%)
	Controls	16 (53.3%)	31 (46.3%)	7 (43.8%)	11 (45.8%)	65 (47.4%)
	Total	30 (100%)	67 (100%)	16 (100%)	24 (100%)	137 (100%)
Follow-up	Patients	13 (44.8%)	34 (54.0%)	9 (56.3%)	10 (47.6%)	66 (51.2%)
	Controls	16 (55.2%)	29 (46.0%)	7 (43.8%)	11 (52.4%)	63 (48.8%)
	Total	29 (100%)	63 (100%)	16 (100%)	21 (100%)	129 (100%)

**Fig. 3:**
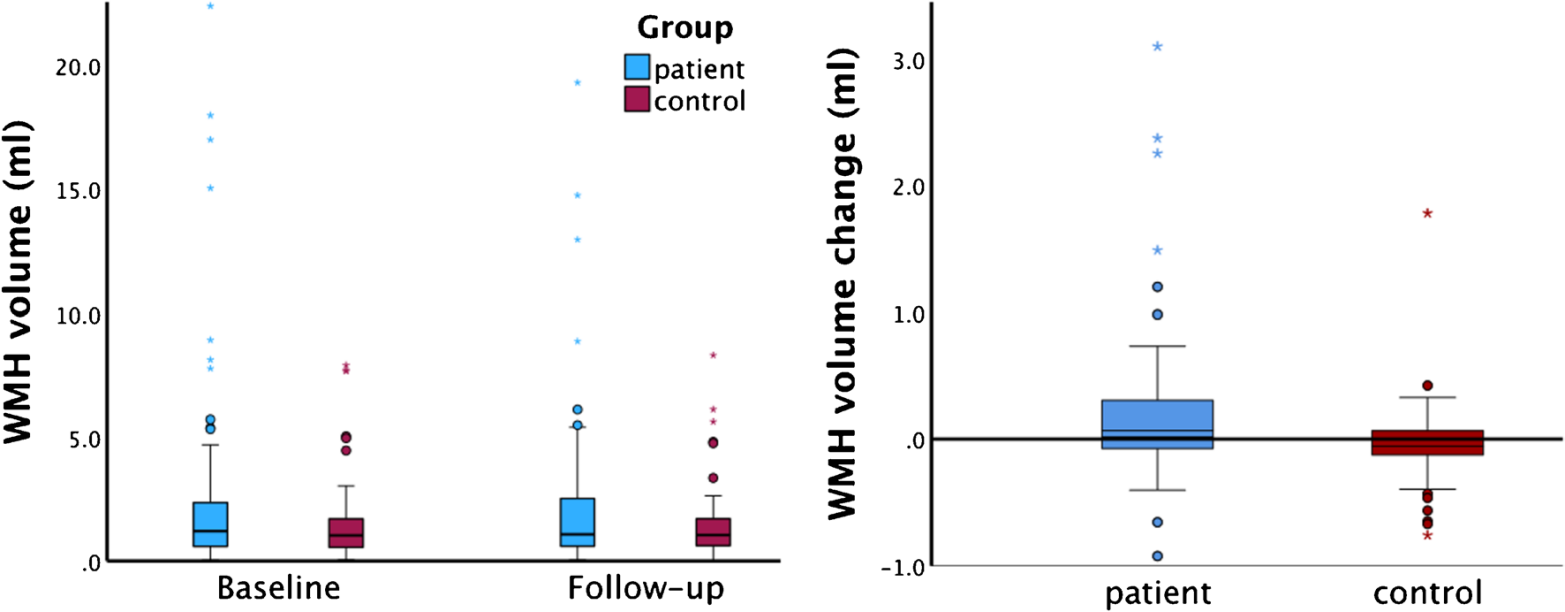
Box plots for the volume data. The left panel shows plots for the WMH volumes at each time point, there was no difference between the patients and controls. The right panel shows the plots for the longitudinal change (baseline – follow-up), and there was a statistically significant group difference with a small WMH volume regression in patients and a small WMH volume progression in controls (*p* = 0.002)

The automatically segmented volumes were used for group analysis, except for minor editing performed in two patients with encephalitis (0.22 ml added) and corpus callosum lesions (0.03 ml added), respectively. There was no difference of total WMH volume between the groups at each time point per se (*p* values 0.19 and 0.55, respectively). However, there was a small difference in change of WMH volume from baseline to follow-up with median volume change 0.136 (95% CI 0.051–0.251) ml higher in patients than controls (*p* = 0.002). The longitudinal change of total WMH volume from baseline to follow-up was a small reduction in patients, and a small increase in controls. Volume of WMHs at baseline and follow-up, as well as the change in volume between the time points, are shown in Table 3. Comprehensive data summary from the Mann–Whitney *U* tests are provided in supplemental material table S1. Box plots for WMH volumes per time point and longitudinal WMH volume change are shown in Fig. 3. Supplemental material figure S1 shows the average lesion pattern for the patients and controls, separately, which serves as a mean of visual comparisons. As it turned out, one center included the majority of participants, and volume data with group comparison from this scanner only is provided in supplemental material table S2. The main results were the same as for the whole study sample.

**Table 3:** Volume of white matter hyperintensities

WMH volume (ml)	Patients^a^	Controls^a^	Difference between medians (95% CI)
Baseline	1.20 (2.63, 1.78, 0.03–22.40)	1.03 (1.51, 1.19, 0.04–7.91)	0.190 (-0.096 – 0.548)
Follow-up	1.07 (2.30, 1.92, 0.03–19.31)	1.05 (1.55, 1.10, 0.01–8.31)	0.089 (-0.194 – 0.402)
Change^b^	0.059 (0.35, 0.37, -0.92–5.02)	-0.057 (- 0.042, 0.19, -0.76–1.78)	0.136 (0.051 – 0.251)

*CI* confidence interval

^a^Values are median (mean, IQR, minimum–maximum)

^b^Subtraction of volumes: Baseline – follow-up

### Other imaging abnormalities

The most prevalent imaging finding was cranial nerve enhancement, and details on frequency, clinical correlation and reliability were reported in a separate cohort study [20]. Imaging findings at baseline are listed in Table 4.

**Table 4:** Imaging findings at baseline

	N of 72 patients	N of 65 controls
Encephalitic pattern	1^b^	0
Mass lesion	0^c^	0
Enhancement^a^ of brain lesion	0^c^	n.a
Leptomeningeal enhancement^a^	2	n.a
Cranial nerve enhancement^a^	39^d^	n.a
White matter lesions	Table 2	Table 2
Incidental findings on study MRI:		
Focal cortical dysplasia	1	0
Old infarct	2	1
Cavernous malformation	1	0
Arachnoid cyst	2	1
Marchiafava-Bignami lesion	1	0
Parotid tumor	2	1
Additional clinical imaging examination^e^:		
CT brain	7	
CT cerebral angiography	2	
CT cerebral venography	1	
MRI brain	7	
MRI cervical spine	2	
MRI lumbar spine	4	
MRI total spine	3	
MRI brachial plexus	1	
MRI sacral plexus	1	

^a^contrast was administered to 69 patients (1 too high and 1 unknown glomerular filtration rate and 1 injector issue), no contrast was administered to controls; ^b^1 eligible encephalitis case declined invitation; ^c^1 case had mass lesions in cervical spinal cord that responded to antibiotic treatment; ^d^Separate substudy [20]; ^e^Findings were: 1 vasculitis, 1 spinal cord mass lesions, 1 myelitis, 1 acute inflammatory demyelinating polyneuropathy, 2 plexus neuritis

**Fig. 4:**
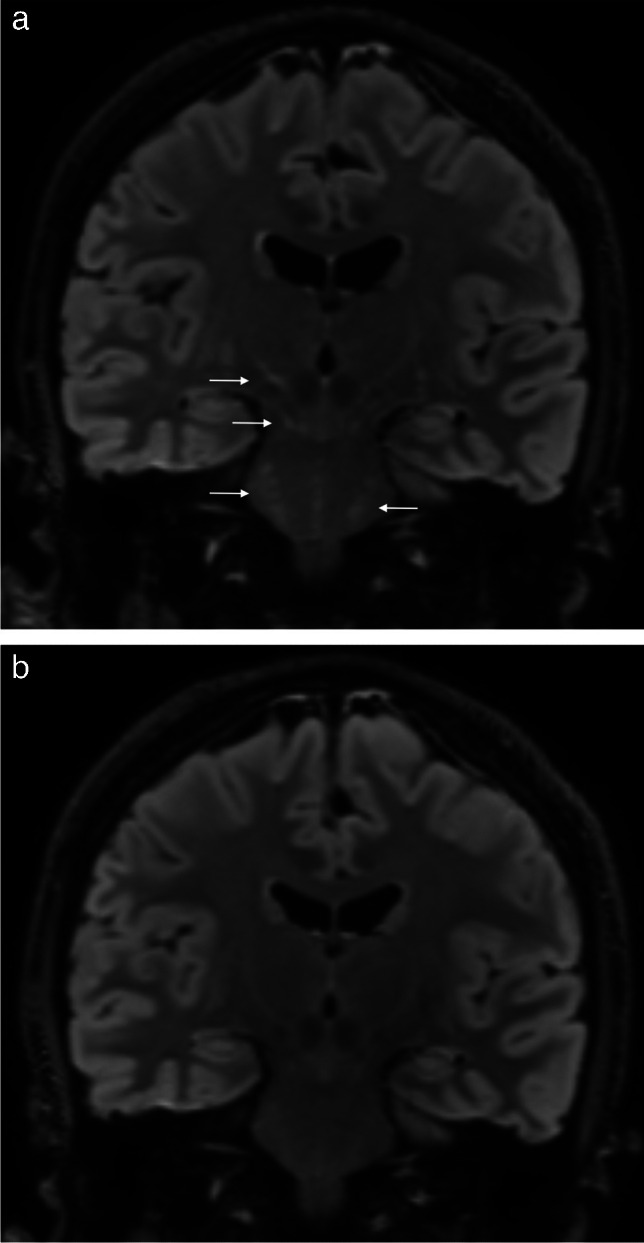
Encephalitis. Coronal FLAIR images from definite neuroborreliosis case with encephalitis showing small hyperintensities (arrows) in right crus cerebri, mesencephalon and pons bilaterally at baseline (**a**) (hyperintense changes were also seen in internal capsules and cerebellar dentate nuclei, not shown here). There was complete regression of the findings at follow-up (**b**)

For all participants except two patients, the WMHs were of non-specific character typically attributed to small vessel disease. One patient with small hyperintensities of internal capsule, crus cerebri, mesencephalon, pons and cerebellar dentate nuclei was considered to have encephalitic pattern (Fig. 4). This patient also had leptomeningeal enhancement, and CT cerebral angiography was consistent with subtle findings of vasculitis. Criteria for definite neuroborreliosis were fulfilled for this patient.

Another patient had two non-enhancing lesions in the corpus callosum at baseline, but not at follow-up (supplemental Fig. 3). The *Bb* antibody index was negative for this patient, so neuroborreliosis diagnosis was not definite and a different infectious etiology cannot be ruled out. However, the CSF was negative for enterovirus, varicella zoster, herpes simplex type 1 and 2 DNA, and for tick borne encephalitis virus specific IgG and IgM. The cell count was 372 and duration of symptoms was nine days, making neuroborreliosis a probable diagnosis in an endemic area.

Important findings related to the infection for other patients that were revealed by additional, clinically initiated imaging examinations were: Cervical spinal cord mass lesions, myelitis, acute inflammatory demyelinating polyneuropathy and plexus neuritis.

### Correlation analysis

For patients, but not for controls, the change in WMH volume showed moderate correlation with the various measures of WMH severity at baseline (WMH volume, count and Fazekas scale grade respective *p* values 0.002, 0.008, and 0.01) and age (*p* = 0.03). There was no correlation with the selected molecular markers (sNfL, CSF cell count and total protein), fatigue or CCS in the patient group. In the control group, increase of WMH volume from baseline to follow-up correlated with baseline fatigue (*p* = 0.02). Table 5 provides the full list of correlation coefficients with CIs and *p* values for the correlation analyses.

**Table 5:** Results of exploratory correlation analyses

Correlation of WMH vol. change (ml)	Patients		Controls	
	Spearman *ρ* (95% CI)	*p* value	Spearman *ρ* (95% CI)	*p* value
WMH vol. baseline	0.39 (0.15–0.58)	0.002*	-0.05 (-0.31–0.22)	0.73
Fazekas baseline	0.31 (0.06–0.52)	0.01*	-0.15 (-0.40–0.12)	0.25
WMH count baseline	0.33 (0.08–0.54)	0.008*	-0.21 (-0.45–0.06)	0.12
Age	0.27 (0.02–0.49)	0.03*	-0.07 (-0.33–0.19)	0.58
sNfL pg/ml	0.20 (-0.09–0.46)	0.17		
CSF cells/mm^3^	0.01 (-0.25–0.26)	0.94		
CSF protein g/l	0.07 (-0.20–0.32)	0.61		
FSS baseline	-0.04 (-0.29–0.22)	0.77	-0.31 (-0.53– -0.05)	0.02*
FSS follow-up	-0.05 (-0.31–0.22)	0.71	-0.16 (-0.42–0.12)	0.25
CCS baseline	0.18 (-0.10–0.43)	0.20		
CCS follow-up	-0.06 (-0.34–0.22)	0.67		

*CCS* composite clinical score, *CSF* cerebrospinal fluid, *CI* confidence interval, *FSS* Fatigue Severity Scale, *sNfL* serum neurofilament light chain, *vol* volume

After controlling for age with hierarchical multiple linear regression, the baseline WMH volume, sNfL and baseline CCS, explained 59% of the variance in WMH volume change, *p* value < 0.001. Only the relationship between baseline WMH volume and volume change was statistically significant with $\beta$ = 0.79, *p* < 0.001 (sNfL $\beta$ = 0.18, *p* = 0.07; CCS baseline $\beta$ = 0.11, *p* = 0.24).

### Reliability

The percentages of agreement between raters for binary scores were: encephalitis 100%, mass lesion and enhancement of brain lesion both 98.6% and enhancement of leptomeninges 97%. Weighted kappa for rating of Fazekas scale and WMH lesion count were 0.66 (95% CI 0.52–0.79) and 0.82 (95% CI 0.72–0.92), indicating substantial and almost perfect agreement [31]. The ICC was 1.00 (95% CI 1.00–1.00) for the volumetry reliability dataset (*N* = 24) with the volumes from automatic segmentation as well as the two raters’ editing of these automatic segmentations, indicating excellent reliability. Median DSCs (IQR) between raters and automatic segmentation was 0.70 (0.18), and 0.68 (0.19), and between raters this coefficient was 0.98 (0.03).

## Discussion

This longitudinal case–control study shows that patients with neuroborreliosis do not have more WMH lesions than healthy controls by visual assessment. However, the temporal evolution in volume of WMHs appears to be different in patients compared to controls. The volume decreased from baseline to follow-up in patients, while it increased slightly in controls, as expected [29, 32, 33]. For patients, more WMH lesions, higher Fazekas scale grade and higher volume of WMHs at baseline were associated with more WMH volume change. These associations were not found in the controls. Cranial nerve enhancement is a common imaging abnormality in neuroborreliosis, while meningeal enhancement, encephalitis and vasculitis are rare. The pattern of main involvement in the brain stem in the case with encephalitis in this cohort, is the same as in the majority of case reports/series [34–44]. Mass lesions or brain enhancement are also rare [45].

The challenge of misdiagnosing neuroborreliosis as multiple sclerosis or vice versa seems to be overstated. The two main systematic imaging studies that contributed to establish such a link have important shortcomings due to small samples, lack of a control group for WMH evaluation, inclusion of patients with diagnosis of relapsing–remitting multiple sclerosis, and only small proportions had intrathecal *Bb* antibody production [4, 10]. Application of serum positivity as an inclusion criterium for studies of neuroborreliosis is a limitation that is underlined by the results from a large French study of 569 patients with suspected borreliosis. It showed that a different diagnosis was found in 41% of the seropositive cases [46]. Also of interest, *Bb* serum positivity is not more frequent in patients with multiple sclerosis than in the healthy population [47–49]. One patient in our study cohort had lesions in the corpus callosum. This was not a case of definite neuroborreliosis according to the established criteria. The patient also presented radicular pain, enhancement of caudal nerve roots and age above 50 years which by consensus are ‘red flags’ that point away from multiple sclerosis [50].

WMHs is an unspecific imaging finding that is common in the middle-aged and elderly population [51]. Although it can occur in demyelinating disorders, infection or neoplastic lesions, the pattern of relatively symmetric and mainly frontoparietal location is typical for WMHs of presumed vascular origin [52]. Together with lacunes, perivascular spaces, microbleeds, recent subcortical infarcts and atrophy, WMHs is an important marker for small vessel disease. The main pathological finding in WMHs compared to normal appearing white matter are myelin pallor and astrocytopathy [53]. However, the role of more transient and reversible factors such as interstitial fluid alterations are gaining attention [54]. Regression of WMH volume can be masked when studying changes over time or looking at population means, so studying non-linearity in development of non-specific WMHs is challenging. A recent systematic review and meta-analysis aimed to study longitudinal changes of WMHs, and found a regression in one third of individuals with small vessel disease [29]. In Alzheimer’s disease, WMH regression was more evident in subjects with high burden of imaging markers for small vessel disease [55]. In a study of the temporal dynamics in cerebral small vessel disease over nine years, no associations with vascular risk factors were found for individuals with regression, but non-linear changes and progression was associated with age and baseline WMH severity [33].

Surprisingly, the results of our study contribute to shed light on the evolution of non-specific WMHs. The patients, unlike controls, had regression of WMH volume from time of infection to follow-up after treatment, pointing to a role of inflammatory pathways in the dynamics of WMHs. Also, we found an association of WMH volume change with severity of WMHs in the patients, but not in controls. This supports the concept of ‘brain frailty’ [56] and ‘brain resilience’ [57], and that small vessel disease constitutes or confounds a risk factor for vulnerability. In neuroborreliosis, it seems that the infection or inflammatory response causes a different or more pronounced effect on small vessels in the brain of patients with such vulnerability. This effect could be increased vessel permeability, or other mechanisms for increased interstitial fluid, and more activation of monocytes and microglia with further enhanced cytokines and inflammation, and possibly transient myelin damage. There was no statistically significant association of WMH volume change with sNfL level, a marker for neuroaxonal damage, which has been reported to be more elevated in neuroborreliosis cases with stroke and central nervous system involvement compared to cases with only peripheral nervous system involvement [58].

In the control group, there was correlation between fatigue at baseline and increase of WMHs, and this is in line with results from a meta-analysis that reported association between fatigue and higher severity of WMH in small vessel disease [59]. This correlation was not found in the patient group, and is likely attributable to the higher degree of fatigue and variations in fatigue related to the infection per se [12].

Among the strengths of this study was the generalizability with a large and prospectively selected sample, where the patients were diagnosed according to established criteria [3]. Also, there was a high agreement and reliability of ratings and volume measurement.

The results are suggestive of a small difference in WMH volume at baseline, albeit the study power was limited due to lower WMH volumes and lower proportions of participants with more severe WMH load compared to other studies [33, 51]. Individuals exposed to ticks are generally active and engaged in outdoor recreation, and are also overall healthier than the general population [60]. The same may apply to the control group, where mobility and motivation are likely among important determinants for participation. The use of two scanners from different vendors introduces technical variability which also influences study power [61]. Image harmonization was part of pre-processing before lesion segmentation to mitigate some of this effect, but no statistical modelling was performed to control for the effect of different scanners. The results of analyses of the dataset from scanner A suggest this limitation is minor.

There was no difference between the groups regarding cognitive function or socioeconomic status [12, 17], but a limitation is the lack of correction for other factors that are known to confound WMH volume such as hypertension, smoking and diabetes [54]. The study was carried out in a Norwegian sample, and there is some limitation in generalizability due to geographical differences in the* Bb* species and strains present in the ticks.

This study did not find more WMH lesions in patients with neuroborreliosis compared to controls. Rare cases of encephalitis or focal lesions can be encountered, but these are readily recognized due to different spatial distribution. The study results, however, suggest that neuroborreliosis induces a small temporary increase of total WMH volume in individuals with higher degrees of baseline WMH severity, without association with burden or outcome of the infection. Overall, non-specific WMHs on brain MRI should not be considered suggestive of neuroborreliosis.

## Supplementary Information

Below is the link to the electronic supplementary material.

Supplementary file1 (DOCX 7.71 MB)

## Data Availability

Anonymized data not published within this article will be made available by request from any qualified investigator.
