# Supplementary material for: Are white matter hyperintensities associated with neuroborreliosis? The answer is twofold
Source: Neuroradiology. 2024 Oct 18;67(1):37–48. doi: 10.1007/s00234-024-03482-0 (PMC11802615; doi:10.1007/s00234-024-03482-0)

Neuroradiology Supplemental material

# Are white matter hyperintensities associated with neuroborreliosis? The answer is twofold

*Authors:*

Elisabeth S. Lindland^1,2^, Martin S. Røvang^3^, Anne Marit Solheim^4,5^, Silje Andreassen^2,6^, Ingerid Skarstein^5,7^, Nazeer Dareez^1^, Bradley J. MacIntosh^3^, Randi Eikeland^8,9^, Unn Ljøstad^4,5^, Åse Mygland^4,5^, Steffan D. Bos^7,10,11^, Elling Ulvestad^5,7^, Harald Reiso^8^, Åslaug R. Lorentzen^4,8^, Hanne F. Harbo^2,11^, Atle Bjørnerud^12^, Mona K. Beyer^2,13^

*Affiliations:*

^1^Department of Radiology, Sorlandet Hospital, Arendal, Norway, ^2^Institute of Clinical Medicine, University of Oslo, Oslo, Norway, ^3^Department of Physics and Computational Radiology, Oslo University Hospital, Oslo, Norway, ^4^Department of Neurology, Sorlandet Hospital, Kristiansand, Norway, ^5^Institute of Clinical Medicine, University of Bergen, Bergen, Norway, ^6^Department of Pediatrics, Sorlandet Hospital, Arendal, Norway, ^7^Department of Microbiology, Haukeland University Hospital, Bergen, Norway, ^8^The Norwegian National Advisory Unit on Tick-borne Diseases, Sorlandet Hospital, Norway, ^9^Faculty of Health and Sport Sciences, University of Agder, Kristiansand, Norway, ^10^Cancer Registry of Norway, The Norwegian Institute of Public Health, Oslo, Norway, ^11^Department of Neurology, Oslo University Hospital, Oslo, Norway, ^12^Department of Physics, University of Oslo, Oslo, Norway, ^13^Division of Radiology and Nuclear Medicine, Oslo University Hospital, Oslo, Norway

Corresponding author: Elisabeth S. Lindland, e-mail: Elisabeth.M.S.Lindland@sshf.no

**Supplemental Table S1.** Values from the Mann-Whitney *U* test

| Hypothesis | Group | N | Mean rank | U | Z | p value | r |
| --- | --- | --- | --- | --- | --- | --- | --- |
| Baseline WMH volume is different in patients vs. controls | Patients  Controls | 72  64 | 72.70  63.77 | 2001.5 | -1.319 | 0.187 | -0.113 |
| Follow-up WMH volume is different in patients vs. controls | Patients  Controls | 66  60 | 65.38  61.43 | 1856 | -0.606 | 0.547 | -0.054 |
| Change in WMH volume from baseline to follow-up is different in patients vs. controls | Patients  Controls | 64  59 | 71.53  51.66 | 1278 | -3.088 | 0.002 | -0.278 |

WMH - White matter hyperintensity

**Supplemental table S2.** Volume of white matter hyperintensities, data for participants at scanner A. *P* values for the Mann-Whitney *U* tests were 0.054 at baseline, 0.218 at follow-up and 0.005 for the change in volume from baseline to follow-up.

| WMH volume (ml) | Patients (n=53)^a^ | Controls (n=56)^a^ | Difference between medians (95% CI) |
| --- | --- | --- | --- |
| Baseline | 1.32 (1.73, 0.35-22.40) | 1.05 (1.14, 0.26-7.91) | 0.290 (-0.005 – 0.676) |
| Follow-up | 1.18 (1.84, 0.40-19.31) | 1.06 (1.08, 0.26-8.31) | 0.174 (-0.097 – 0.493) |
| Change^b^ | 0.096 (0.46, -0.92-5.02) | -0.051 (0.19, -0.76-1.78) | 0.142 (0.045 – 0.263) |

^a^Values are median (IQR, minimum-maximum).

^b^Subtraction of volumes: Baseline – follow-up

CI – confidence interval

**Supplemental table S3.** Contingency tables for visual rating of WMHs with Fazekas scale at baseline and follow-up. The distribution of proportions did not differ between the groups: Baseline χ^2^ = 1.48, *p*= 0.48, Cramer’s V = 0.10, and follow-up χ^2^ = 0.042, *p*= 0.98, Cramer’s V = 0.018 (in both tests the two highest levels were combined because the original table shown here has 25% of cells with expected count <5).

| Fazekas scale | | Absent | Punctate foci | Beginning confluence | Large confluent areas | Total |
| --- | --- | --- | --- | --- | --- | --- |
| Baseline | Patients | 25 (46.3%) | 38 (55.9%) | 6 (50.0%) | 3 (100%) | 72 (52.6%) |
|  | Controls | 29 (53.7%) | 30 (44.1%) | 6 (50.0%) | 0 (0%) | 65 (47.4%) |
|  | Total | 54 (100%) | 68 (100%) | 12 (100%) | 24 (100%) | 137 (100%) |
| Follow-up | Patients | 28 (50.9%) | 31 (50.8%) | 6 (50.0%) | 1 (100%) | 66 (51.2%) |
|  | Controls | 27 (49.1%) | 30 (49.2%) | 6 (50.0%) | 0 (0%) | 63 (48.8%) |
|  | Total | 55 (100%) | 61 (100%) | 12 (100%) | 1 (100%) | 129 (100%) |

**Supplemental figure S1.** Lesion frequency maps superimposed onto a standard brain template shows the average lesion pattern for the controls (left panel) and patients (right panel) separately, and for each time point (baseline in upper panel, follow-up in lower panel). The color-coded scale represents percentage of population with voxels classified as WMH lesion. It shows that lesion distribution is mainly frontoparietal, periventricular and symmetric, which is consistent with the pattern seen in non-specific changes typically attributed to vascular origin.


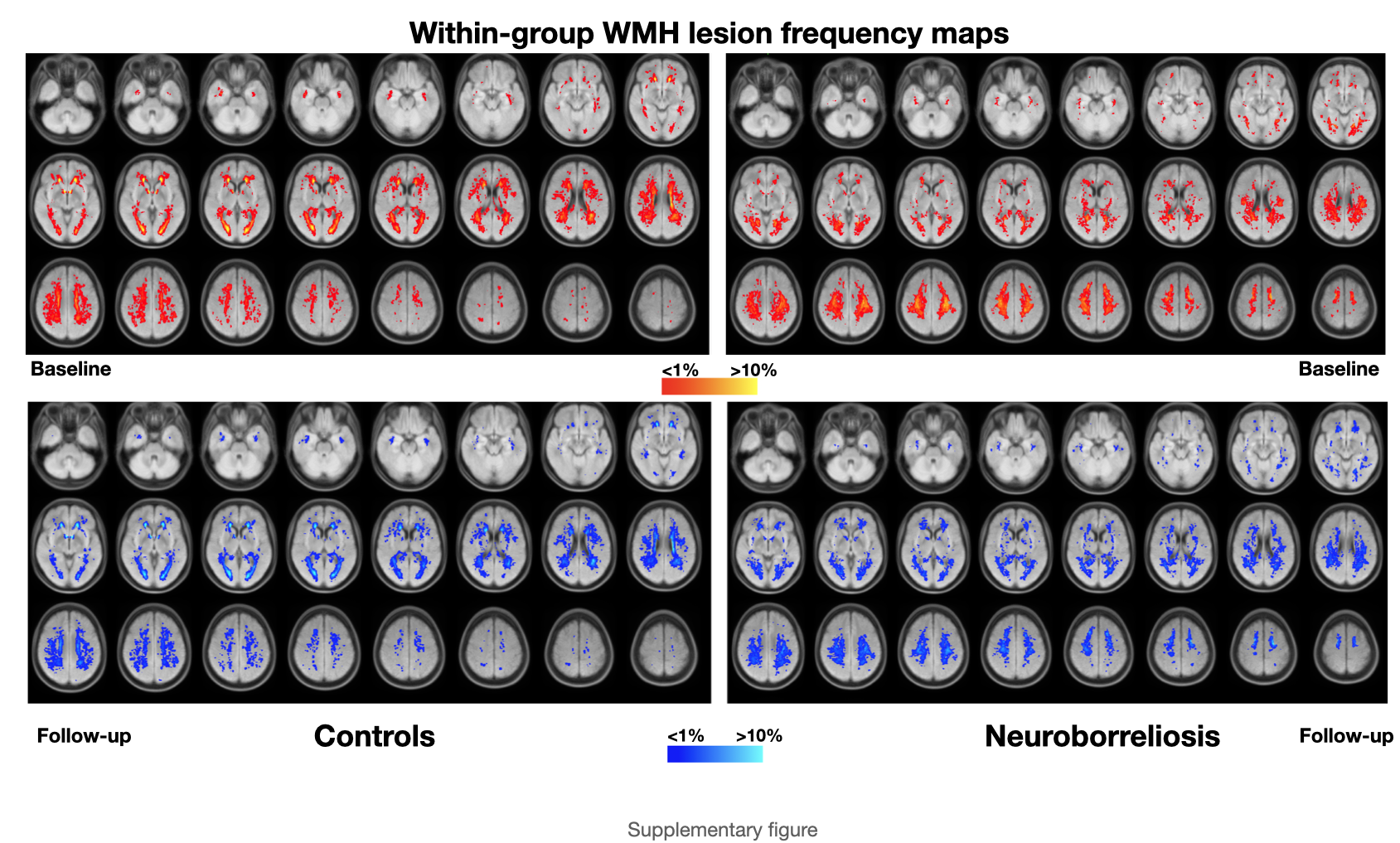


**Supplemental figure S2.** Sagittal FLAIR images from a probable neuroborreliosis case, diagnosis was not definite due to lack of intrathecal production of *Bb* specific antibodies. There were two hyperintense lesions of corpus callosum (left image), one in genu (arrow) and one in body (arrowhead). The most posterior lesion is not fully included in this image slice. There was complete regression of the lesions at follow-up (right image).


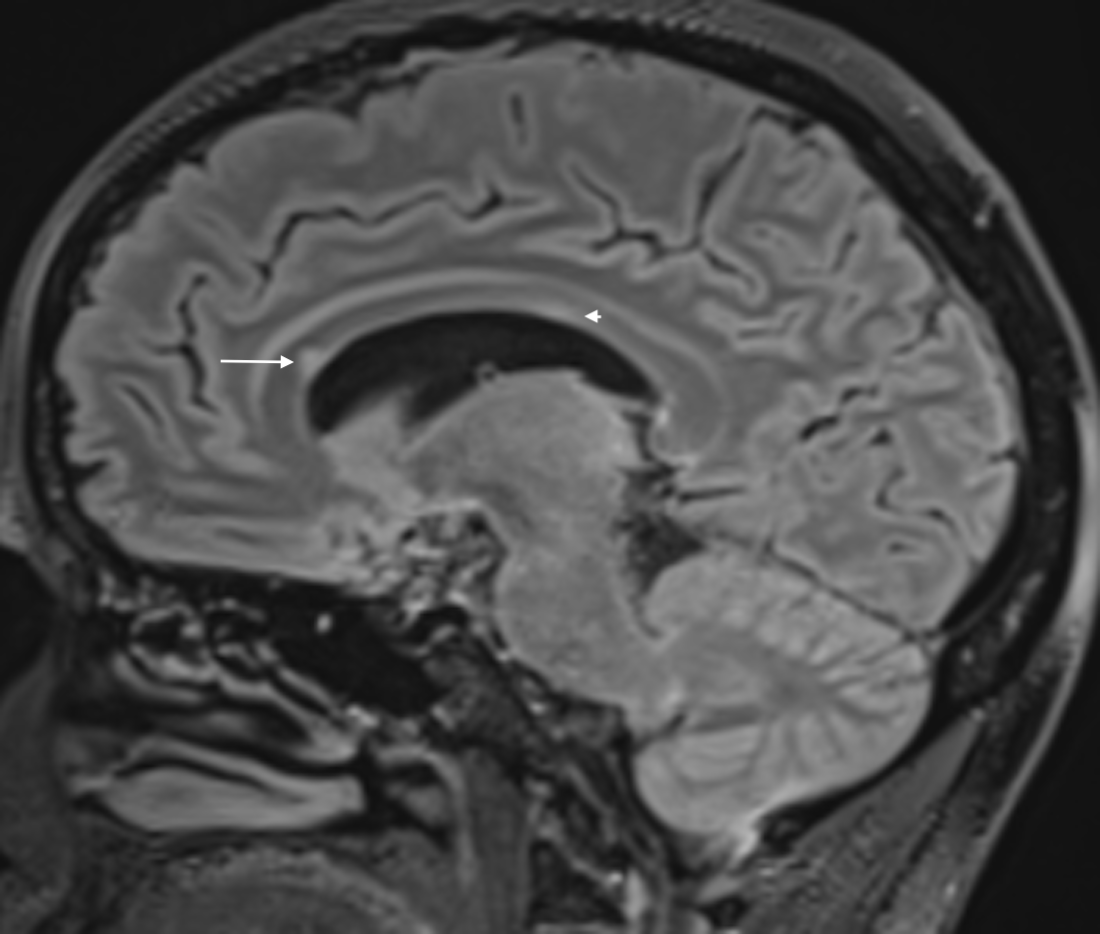

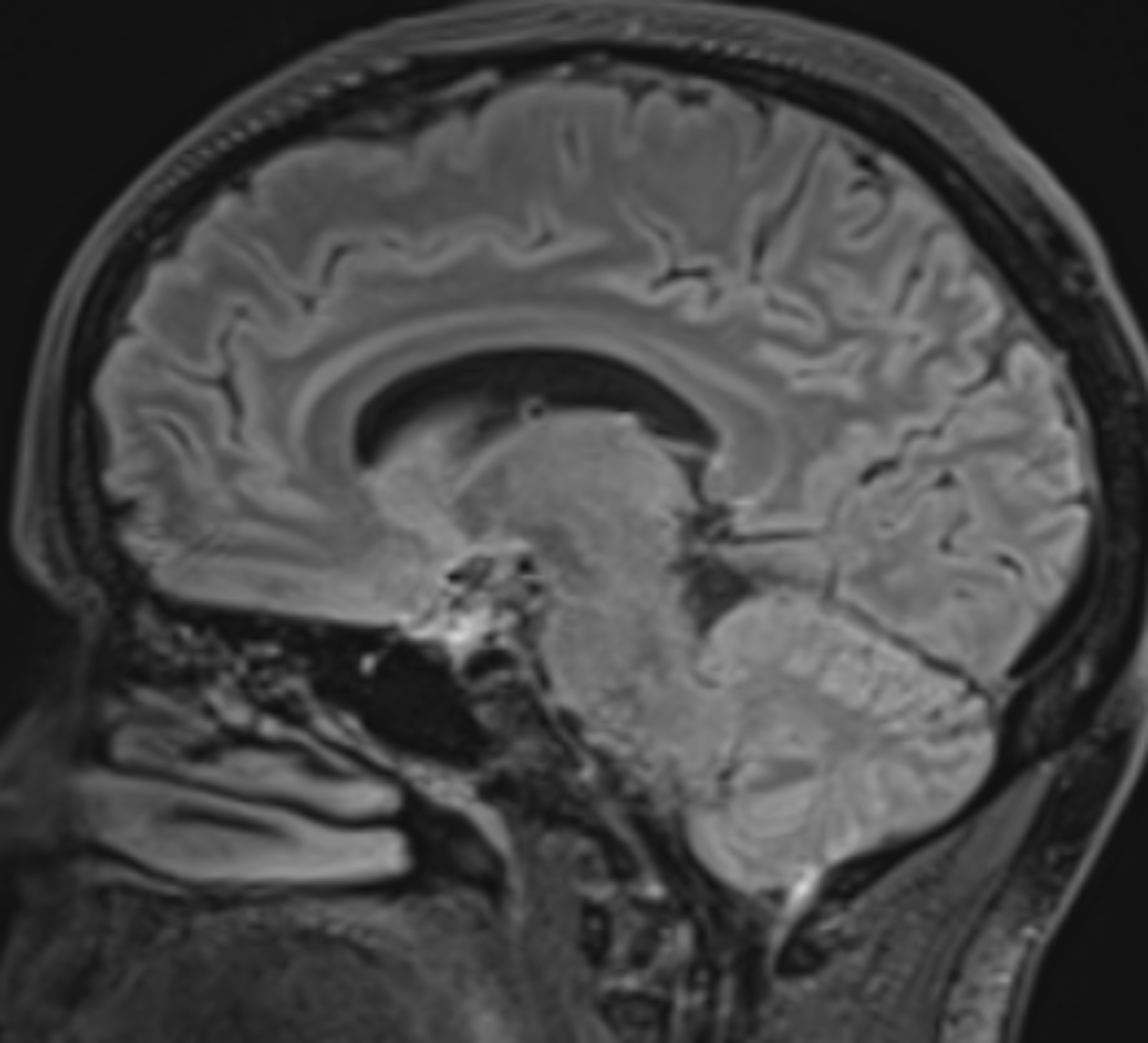

Supplement: Supplementary file 1 — Supplementary file1 (DOCX 7.71 MB) [file 234_2024_3482_MOESM1_ESM.docx]
